# Anatomy and histomorphology of the flexor digitorum profundus enthesis: functional implications for tissue engineering and surgery

**DOI:** 10.1186/s12891-021-04922-1

**Published:** 2021-12-10

**Authors:** Jeremy W. Mortimer, Hamad Alsaykhan, Subashan Vadibeler, Philippa A. Rust, Jennifer Z. Paxton

**Affiliations:** 1 Anatomy@Edinburgh, Deanery of Biomedical Sciences, University of Edinburgh, Old Medical School, Teviot Place, Edinburgh, EH8 9AG UK; 2 Hooper Hand Unit, St John’s Hospital, Livingston, Edinburgh, UK

**Keywords:** Flexor digitorum profundus, Enthesis, Anatomy, Histology, Interfacial tissue engineering

## Abstract

**Background:**

The enthesis possesses morphological adaptations across the soft-hard tissue junction which are not fully restored during surgical avulsion repairs. This loss of anatomical structure, highly related to function, contributes to poor clinical outcomes. Investigating the native macro- and micro-structure of a specific enthesis can provide functional and biomechanical insights to develop specialised, novel tissue-engineered therapeutic options and potentially improve current surgical treatments for avulsion injuries.

**Methods:**

This study examines the anatomy and histomorphology of the flexor digitorum profundus (FDP) enthesis in 96 fresh-frozen human cadaveric fingers, quantitatively and qualitatively analyzing the shape, size, angle of tendon fibres and histological architecture, and explores differences in sex, finger and distance along the enthesis using linear mixed effects models.

**Results:**

Macroscopically, results showed a consistent trapezoidal insertion shape of 29.29 ± 2.35 mm^2^ mean surface area, but with significant morphometric size differences influenced primarily by the smaller dimensions of the little finger. Microscopically, a fibrocartilaginous enthesis was apparent with a 30.05 ± 0.72^o^ mean angle of inserting tendon fibres, although regional variation in fibrocartilage and the angle change of tendon fibres before insertion existed.

**Conclusions:**

The implication of these findings on native and specific FDP enthesis function is discussed whilst providing recommendations for optimal FDP enthesis recreation for interfacial tissue engineers and hand surgeons. The study emphasizes the importance of region-specific knowledge whilst also describing methods applicable to assessing any soft tissue insertion.

**Supplementary Information:**

The online version contains supplementary material available at 10.1186/s12891-021-04922-1.

## Background

The flexor digitorum profundus (FDP) tendon inserts into the base of the distal phalanx (DP) in the finger, functioning to provide a full fist for power grip and fingertip pinch required for everyday manual tasks. FDP avulsion from this insertion (‘jersey finger’) is a common, distinct clinical injury [1, 2] and is the most frequent type of closed flexor tendon injury [3–5]. Such injuries have an extensive economic and social impact, both for the individual and society, due to reliance on effective manual function for work and daily living [6, 7]. Multiple surgical techniques are employed to restore the FDP tendon-bone interface, primarily based on pull-out suture or bone anchor methods, but are at risk of complications such as infection, nailplate deformity, osteolysis and injurious anchor placement [8], contributing to poor functional outcomes [1, 9, 10]. Furthermore, serious infective complications or complete mechanical failure of the reattachment technique require a tendon graft to restore function, with additional patient morbidity and cost. By advancing morphological understanding of the FDP insertion, both the efficacy of current techniques can be increased and new therapeutic options developed using novel tissue-engineered techniques.

The enthesis is the region of soft tissue (e.g. tendon) attachment to bone, allowing transmission of tensile force whilst providing anchorage and dissipation of stress forces between biomechanically distinct tissues [11–13]. Macroscopically, tendons flare out at their insertions, demonstrating the importance of size of interfacial surface area contact for strong attachment and stress dispersal [12, 14]. Microscopically, the majority of entheses also possess an interfacial fibrocartilage transition, providing a gradation in tissue properties [15, 16]. Such entheses are categorized as ‘fibrocartilaginous’ and encompass 4 distinct tissue zones: dense fibrous connective tissue, uncalcified fibrocartilage (UF), calcified fibrocartilage (CF), and bone [17–19]; ‘fibrous’ entheses lack fibrocartilage. Surgical reattachment of an avulsed tendon does not recapitulate the fibrocartilaginous transition zone [20–23], leaving a biomechanically inferior interface [20, 22, 24] that promotes re-rupture risk and poor outcome.

An important structural and biomechanical consideration at the enthesis is tendon fibre angle. A more acute attachment angle increases strain concentration at the enthesis [25] and the change in tendon fibre angle during functional tendon-bone movement generates compressive and shear forces [18]. The fibrocartilaginous enthesis has indeed been portrayed as an adaptation to counter these forces [18], with the quantity of UF associated with a large degree of movement of inserting tendon fibres [18, 26–28]. Therefore, knowledge of tendon fibre orientation at the FDP enthesis may provide biomechanical insights into function and failure, and enhance the surgical recreation, or indeed in vitro formation, of this native anatomical structure.

Interfacial tissue engineering (ITE) aims to establish connecting interfaces between distinct tissues in vitro, and has great potential to provide novel, enhanced therapeutic options for avulsion injuries such as at the FDP insertion described above. By pre-forming a replica enthesis, a surgically implanted model requires integration of only homogenous engineered and native tendon and bone tissue, rather than the heterogenous structures at the complex but vital interface. To realise translational potential, ITE must be specific to a body region, and development of an ITE FDP model demands detailed understanding of FDP enthesis macro- and micro-anatomy. The human FDP enthesis has received little attention, particularly compared to entheses around large joints. Studies regarding its insertional anatomy are limited to position on the DP [29] and vasculature [30], whilst histologically it is mentioned only as part of broad surveys of numerous enthesis sites [31–34]. This study therefore addresses the FDP enthesis anatomy crucial to designing a clinically relevant ITE model, using techniques applicable to any enthesis.

The overall aim of this study was to analyze the FDP enthesis in macroscopic and microscopic detail, focusing on shape, size, and tendon fibre angle. The specific aims were to: 1) gain insight into biomechanical functionality, 2) aid translational ITE design, and 3) enhance knowledge relevant to current surgical repair techniques of the avulsed tendon. Both qualitative and quantitative analyses were performed, with morphometric variation explored through comparison of different sexes, fingers and distances along the enthesis.

## Methods

### Tissue procurement and dissection

A retrospective cohort study of type III evidence was performed on fresh-frozen human cadaveric tissue, obtained from The University of Edinburgh Medical School body donation programme and regulated by The Human Tissue (Scotland) Act (2006). All donors consented to photography before death.

All 96 fingers from 12 donors were dissected for either footprint (3 male donors, 3 female; mean age 82.2, range 65–95) or histological (3 male, 3 female; mean age 79.3, range 73–91) investigation, selected as a sample of convenience but to provide equal sex balance. 3x magnification was used throughout dissection, and no gross pathology or previous surgery of the FDP insertion was observed. Fingers from both study groups underwent the same initial dissection to obtain an isolated FDP-DP tendon-bone sample: from a midline flexor approach, all appreciable soft tissue was sharply removed from the DP except for an approximate 5 cm length of FDP. All components of the distal interphalangeal joint (DIPJ) capsule were carefully excised with avoidance of FDP insertion disruption.

### Insertion footprint analysis

**Fig. 1:**
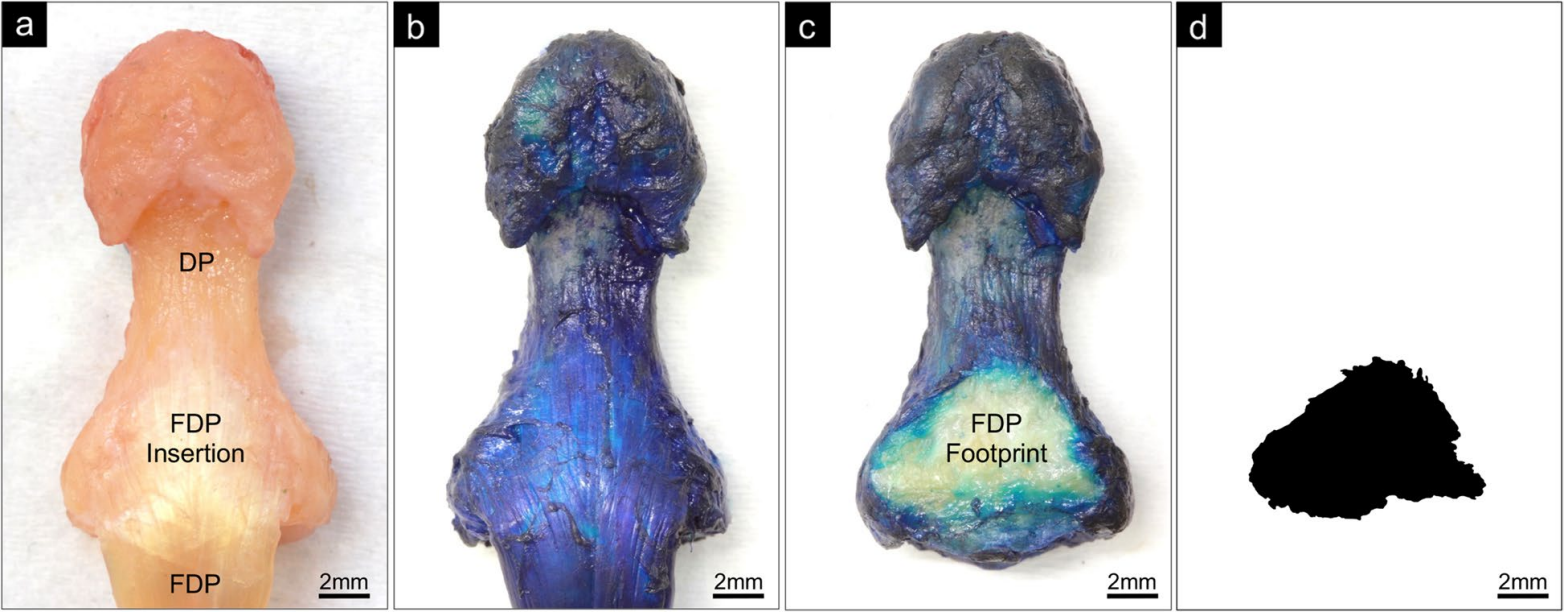
FDP Footprint Generation. **a**) Excised distal phalanx (DP) bone with attached FDP tendon (flexor surface). **b**) Tendon-bone sample after methylene blue immersion. **c**) DP after sharp excision of the FDP at insertion, leaving the unstained FDP footprint. **d**) Binary mask image of the FDP footprint for morphometric analysis ​

Shape and size of the FDP insertion were assessed by revealing the tendon footprint on the bone, adapting and enhancing a previously published inking methodology [29]. The isolated FDP-DP sample was immersed in methylene blue 1% w/v aqueous solution (Scientific Laboratory Supplies Ltd., Coatbridge, UK) for 10 s, and excess ink briefly blotted away on removal (Fig. 1a, b). The FDP was then sharply dissected away at the bone-tendon interface, leaving the unstained FDP footprint (Fig. 1c). After drying at room temperature (1 h), a digital photograph of the unstained FDP footprint was captured for 2-dimensional (2-D) image measurements. During optimisation of the inking timeframe, one sample underwent 90 min immersion, noticeably reducing the footprint size, and was therefore excluded from further analysis, leaving a sample size of 47 footprint images.

Image analysis of digital photographs was conducted using ImageJ software (National Institutes of Health, Bethesda, MD). The FDP footprint perimeter was manually outlined at the demarcation of peripheral colour change, at the point where variation from the dark blue of the methylene blue was first perceived, and subsequently processed into a binary mask image (Fig. 1d). Footprint surface area was calculated from the mask image, whilst a software-generated bounding box applied to the mask image perimeter allowed precise measurements of the height, base width, apex width and 4 principal internal angles of the footprint shape (Fig. S1). A 2nd observer undertook all footprint measurements on the original unprocessed photographs using the same image analysis technique.

### Histological analysis

Isolated tendon-bone samples for histological observation were further trimmed by excision of their ungual tuberosities and shortening of the attached FDP to approximately 5 mm. Samples were immediately fixed in 10% neutral buffered formalin (Merck Life Science, Gillingham, UK) for 48 h at 4 °C, then decalcified in Decalcifying Solution-Lite (Merck Life Science, Gillingham, UK) for 72 h at room temperature with gentle rocking and 24 h solution changes. Decalcified samples underwent standard machine processing and manual embedding in paraffin wax, following which para-sagittal 10 μm sections were cut to the longitudinal axis and floated onto standard glass slides, dried at 37 °C before staining. Sections were collected across the central 800 μm in the mid-sagittal plane of the enthesis, calculated using pre-analyzed data of the mean FDP footprint base width for a particular finger and sex. Sections were stained in haematoxylin and eosin (H + E) (qualitative overview analysis) and 0.1% toluidine blue (qualitative and quantitative analysis), and high resolution images of entire sections acquired using a Nanozoomer XR slide scanner (Hamamatsu, Welwyn Garden City, UK).

**Fig. 2:**
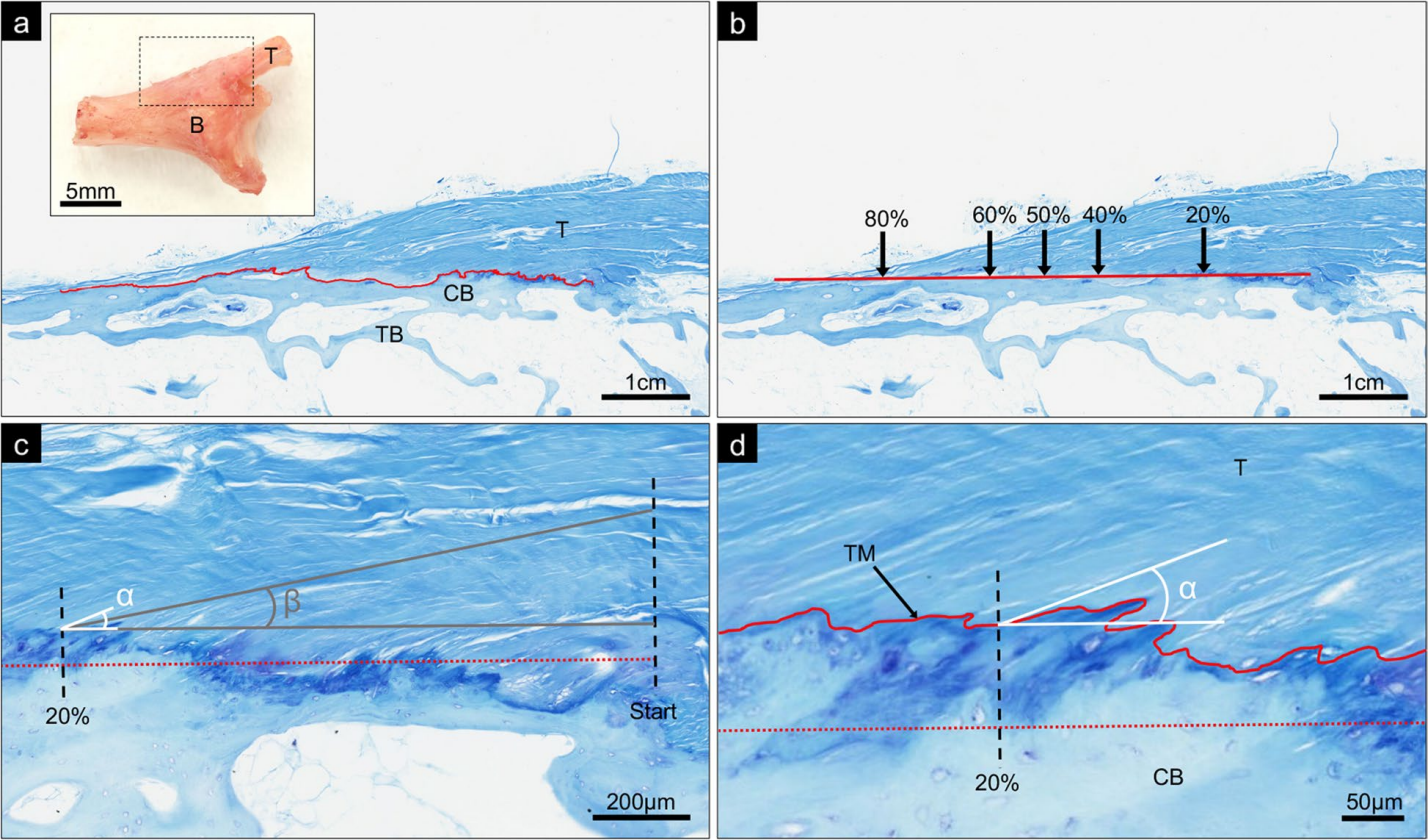
Tendon Fibre Angle Measurement Methodology. **a**) *Inset*: Side view of an example pre-sectioned tendon(*T*)-bone(*B*) sample [ungual tuberosity removed from the distal bone end (*left* side of image)], with box showing enthesis region of main panel. *Main panel*: The tidemark (*superimposed line*) is traced along the length of the FDP enthesis, from proximal (*right*) to distal (*left*). **b**) A line of best fit of the tidemark is produced, marking the points at *20*, *40*, *50*, *60* and *80%* along the enthesis. **c**) Example measurements of angle of tidemark intersection fibres (*α*) and angle of approaching fibres (*β*) at 20% along the enthesis. The location of 20% along the tidemark is found on a perpendicular line (*broken line*) from 20% along the line of best fit of the tidemark (*dotted line*). The angle of tidemark intersection (*α*) measures the angle of directly intersecting fibres; the angle of approach (*β*) measures a line parallel to the average angle of fibres approaching over a preceding 20% distance of the enthesis. Angles are measured against a line parallel to the line of best fit of the tidemark (*dotted line*). **d**) Magnified view to highlight the angle of tidemark (*TM*) intersection (*α*) at this 20% enthesis measurement point. Mid-sagittal section micrographs of an FDP enthesis, toluidine blue. *CB* - cortical bone; *TB* - trabecular bone

Qualitative analysis examined sections for tissue structure and overall configuration of tendon fibres at the enthesis. Quantitative tendon fibre angle measurements were performed on a single toluidine blue stained section on one slide per tendon-bone sample, selected by random number generation. One slide was discovered to contain crumpled sections not allowing representative assessment of tendon fibre angles, and after its exclusion the remaining sample size for quantitative analysis numbered 47 entheses. Angle measurements were performed adapting a previously published method using Image J [25, 35], defining the angle situated between a line parallel with the FDP tendon fibres and a line of best fit of the enthesis tidemark. Both the angle of fibres intersecting the tidemark (*‘inserting fibres’*) and fibres running over a preceding 20% length of the enthesis before reaching the tidemark (*‘approaching fibres’*) were measured at 5 enthesis distance points (20, 40, 50, 60 and 80% along the proximal-distal length) (Fig. 2). ‘*Angle change*’ was defined as the difference in angle between approaching and inserting fibres. All angle measurements were repeated by a 2nd observer on the original blank section images using the same methodology.

### Statistical analysis

Statistical tests were performed in SPSS (version 24; IBM, Armonk, NY). Linear mixed effects models were employed to account for correlation of samples from the fingers of the same donor, where donor identification was defined as a random effect and output generated estimated marginal means and standard errors in all models. A series of models tested hypotheses of the size effect of different sex, finger and individual finger (classified by both sex and finger), as fixed factors testing main effects, for both footprint and tendon fibre angle analysis. Further models tested side of base and apex internal footprint angle and enthesis distance measurement point as additional fixed factors, for footprint shape and tendon fibre angle analysis, respectively. An alpha level of 0.05 was set, and a Bonferroni post-hoc correction applied. Reliability of measurements was ascertained by the intraclass correlation coefficient (ICC) of single measures of absolute agreement between the 2 observers, presented with 95% confidence intervals.

## Results

### FDP footprint Morphometrics

**Fig. 3:**
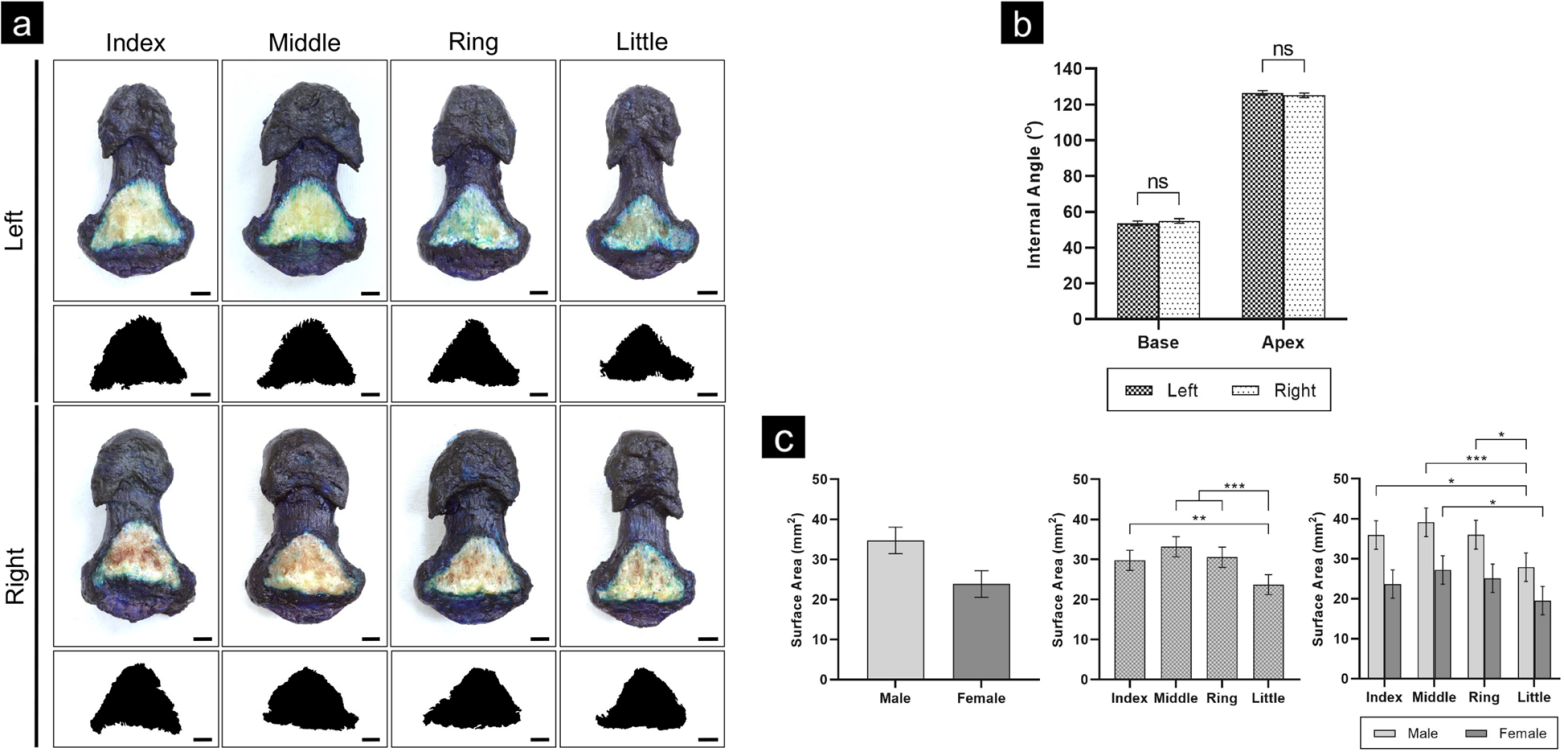
FDP Footprint Shape and Size. Complete set of unstained FDP footprints on stained DPs, with each binary ‘mask’ footprint below, showing trapezoidal footprint shape. Scale bars 2 mm. **b**) Comparison of footprint trapezoid left and right internal angles. Non-significant (*ns*) differences suggest a symmetrical shape across the vertical axis. **c**) Footprint surface area, compared within sex and finger categories. The little finger is the principle source of significant size differences. Mean ± standard error. *p < 0.05, **p < 0.01, ***p < 0.001

The FDP insertion footprint was a consistent shape, approximately trapezoidal and almost triangular (Fig. 3a). A flat, wide base narrowed distally to a more variable flat or rounded apex. Left and right internal trapezoid angles were similar, implying a symmetrical footprint shape (Fig. 3b, Table S-1). Overall mean surface area of insertion was 29.29 ± 2.35 mm^2^. As an indication of general size differences, surface area ranged from the female little finger (19.50 ± 3.56 mm^2^) to male middle (39.11 ± 3.56 mm^2^), with little finger surface area significantly smaller than all other fingers both with sexes combined (index, *p* < 0.01; middle and ring, *p* < 0.001) and within males (index and ring, *p* < 0.01; middle, *p* < 0.001), and significantly smaller than the middle finger within females (*p* < 0.05) (Fig. 3c). ICCs for internal angle and surface area measurements were 0.99 (0.990–0.993) and 0.97 (0.95–0.98), respectively.

Overall mean height, base width and apex width of the footprint were 5.45 ± 0.21 mm, 8.58 ± 0.37 mm, and 1.60 ± 0.11 mm, respectively, with individual finger means and combined means for sex and finger reported in Table 1. For height measurements [ICC 0.81 (0.72–0.87)], the little finger was significantly shorter than all other fingers for both combined sex (all *p* < 0.001) and within males (index, *p* < 0.01; middle, *p* < 0.001; ring, *p* < 0.05), with females also significantly shorter than males overall (*p* < 0.05). For base width [ICC 0.85 (0.74–0.91)], the little finger was significantly narrower than middle (*p* < 0.001) and ring fingers (*p* < 0.05), with the index also significantly narrower than the middle (*p* < 0.05), for combined sex, and within males the little finger was significantly narrower than the middle (*p* < 0.05). No significant differences were found for apex width [ICC 0.40 (0.04–0.65)] between or within finger or sex groupings.

**Table 1:** FDP Footprint Morphometrics (mm)

Finger	Height			Base Width			Apex Width		
	Male	Female	***All***	Male	Female	***All***	Male	Female	***All***
**Index**	6.51 (0.34)	4.95 (0.34)	5.73 (0.24)	9.04 (0.56)	7.79 (0.56)	8.41 (0.40)	1.72 (0.27)	1.44 (0.27)	1.58 (0.20)
**Middle**	6.59 (0.34)	4.86 (0.34)	5.73 (0.24)	9.77 (0.56)	8.62 (0.56)	9.20 (0.40)	1.42 (0.27)	2.11 (0.27)	1.77 (0.20)
**Ring**	6.24 (0.36)	4.97 (0.34)	5.61 (0.25)	9.44 (0.58)	8.11 (0.56)	8.77 (0.40)	1.83 (0.29)	1.33 (0.27)	1.56 (0.20)
**Little**	5.20 (0.34)	4.23 (0.34)	4.72 (0.24)	8.46 (0.56)	7.46 (0.56)	7.96 (0.40)	1.37 (0.27)	1.66 (0.27)	1.52 (0.20)
***All***	6.14 (0.30)	4.75 (0.29)	5.45 (0.21)	9.18 (0.52)	7.99 (0.52)	8.58 (0.37)	1.57 (0.16)	1.63 (0.15)	1.60 (0.11)

Mean (± standard error)

### Qualitative Histomorphology

**Fig. 4:**
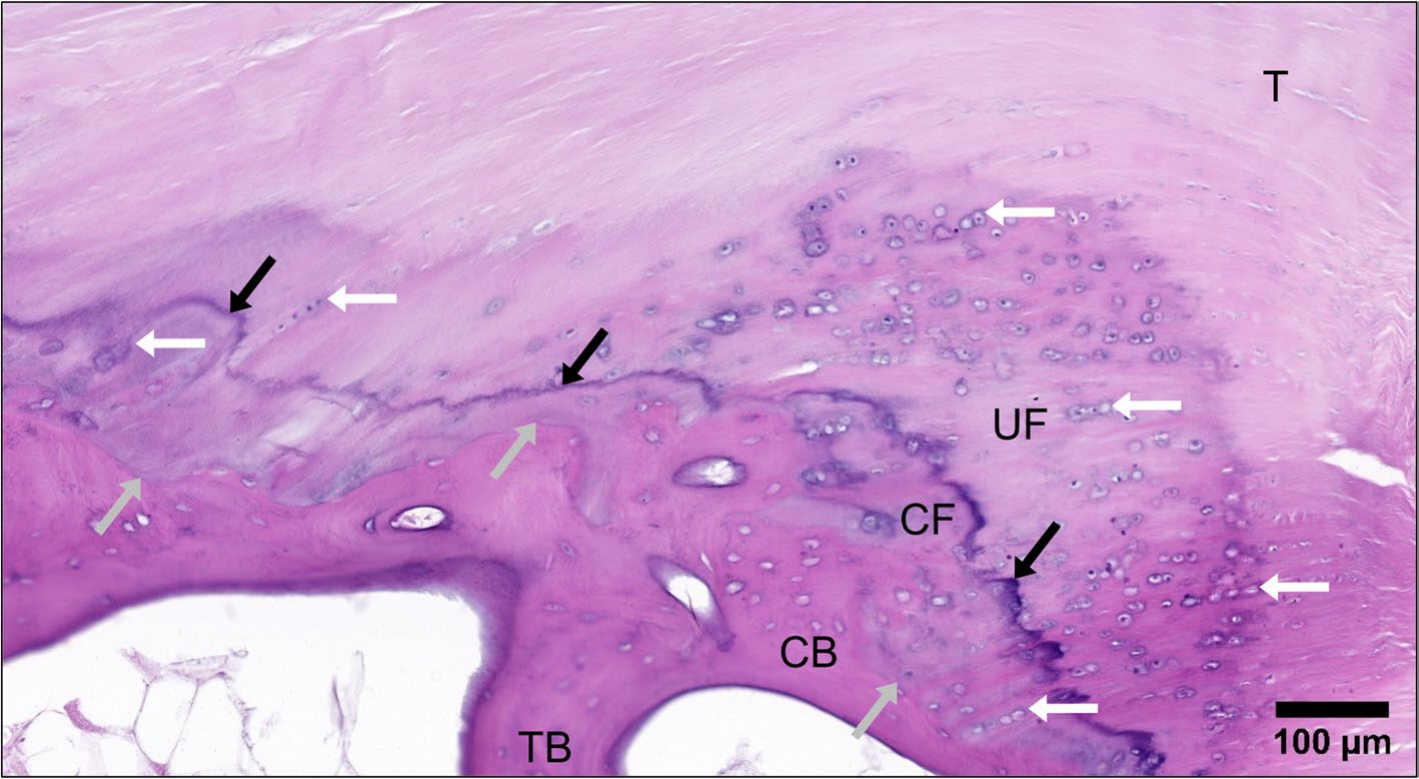
The Fibrocartilaginous FDP Enthesis. The FDP insertion demonstrates the 4 zones of a fibrocartilaginous enthesis: tendon (*T*), uncalcified fibrocartilage (*UF*), calcified fibrocartilage (CF) and bone. The cortical bone (*CB*) is as thin as the trabecular bone (*TB*). The calcified fibrocartilage lies between the tidemark (*black arrows*) and the tendon-bone junction (*grey arrows*). Fibrochondrocytes (*white arrows*), rounded and lying in lacunae within cartilage matrix, indicate cartilaginous areas and generally align in rows. Tendon collagen fibre bundles are continuous through the fibrocartilage areas to attach to the cortical bone. Photomicrograph of a typical mid-sagittal section FDP enthesis, H + E

**Fig. 5:**
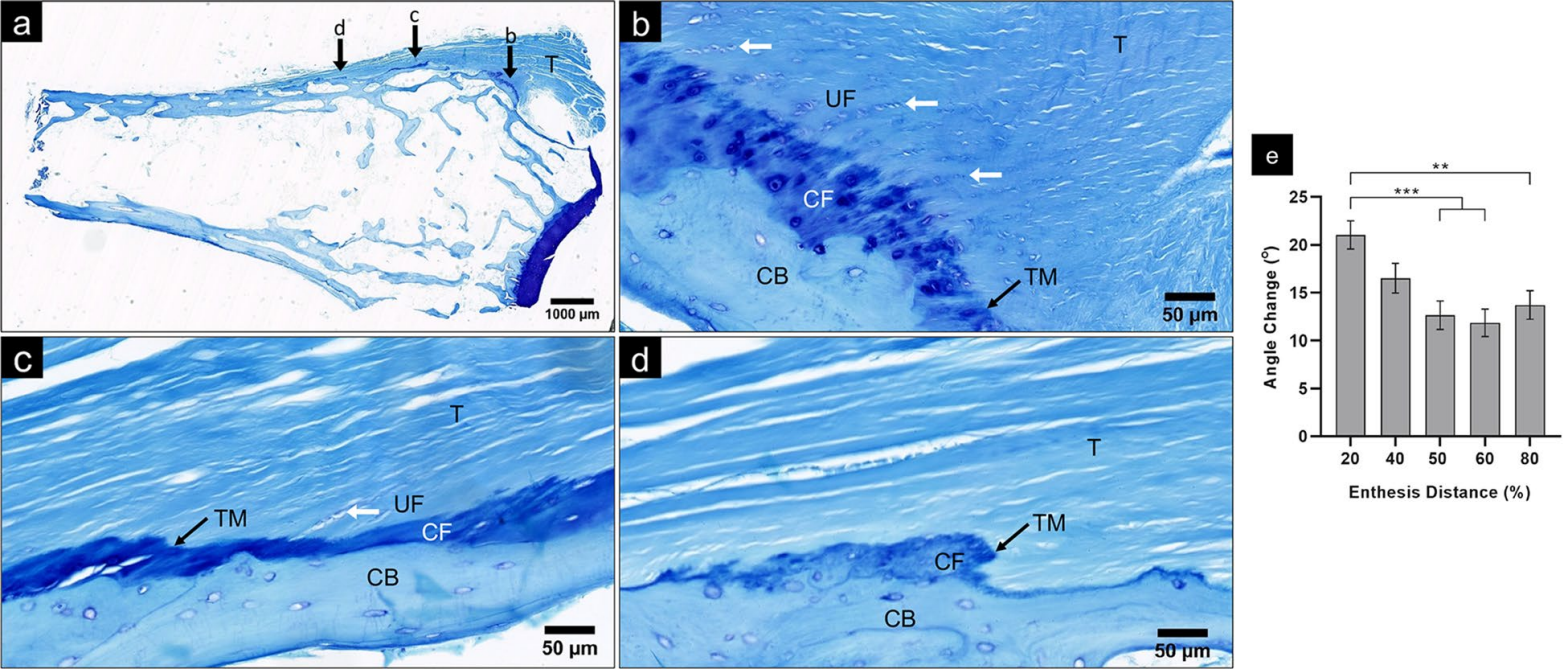
FDP Enthesis Regional Variation. Entire sample section (proximal – *right*; distal – *left*), showing FDP tendon (*T*) attachment; *b*, *c* and *d* indicate subsequent panel regions **b**) Proximal enthesis region. The 4 fibrocartilaginous enthesis zones are apparent. The approaching tendon fibres undergo a considerable angle change in reaching the tidemark (*TM*) and cortical bone (*CB*). The majority of the angle change occurs in the uncalcified fibrocartilage (*UF*) zone, demonstrated by the curved columns of fibrochondrocytes (*white arrows*), with straight tendon fibres in the calcified fibrocartilage (CF) zone. **c**) Middle enthesis region. A calcified fibrocartilage (CF) zone is present, although less thick than in the proximal region, with a variable layer of uncalcified fibrocartilage (*UF*) demonstrated by the limited but perceptible fibrochondrocytes (*white arrow*). Compared to the proximal enthesis region, the approaching tendon fibres are generally less acute to the horizontal, and there is less angle change between the approaching fibres and tidemark intersection fibres. **d**) Distal enthesis region. Areas of calcified fibrocartilage (CF) are sporadic and are interspersed between fibrous enthesis regions which lack any fibrocartilage. The absence of fibrochondrocytes proximal to the calcified fibrocartilage indicates no uncalcified fibrocartilage zone. Tendon fibres approach the tidemark (*TM*) more acutely than the middle enthesis region. Micrographs of a typical mid-sagittal section of an FDP enthesis, toluidine blue (**a**-**d**). **e**) Quantified angle change comparison between the 5 distance measurement points along the thesis. Mean ± standard error **p < 0.01, ***p < 0.001

The FDP enthesis could be classified as fibrocartilaginous, as a fibrocartilage transition between the FDP tendon and DP bone was apparent in all samples (Fig. 4). The fibrocartilage transition was not, however, present throughout the entire enthesis. Considerable enthesis regions contained no fibrocartilage at all, indicating localized fibrous insertion, with some regions possessing only CF without UF. Fibrocartilage, especially UF, predominated in the proximal enthesis region towards the DIPJ (Fig. 5b), becoming less substantial and more sporadic or absent distally (Fig. 5c and d).

The enthesis tidemark commenced as a prolongation of the DIPJ volar plate tidemark and continued distally either between UF and CF zones, or tendon and CF when no UF intervened, and merged with the tendon-bone junction in fibrous regions without fibrocartilage. The dense collagen fibre bundles of the tendon were straight when nearing the tidemark where little or no preceding UF was present (Fig. 5c and d), but curved when traversing regions of abundant UF (Fig. 5b). Tendon fibres did not appreciably deviate as they crossed through the CF zone, maintaining the same angle at the tidemark as at the tendon-bone junction (Fig. 4).

### Quantitative Histomorphology

Tendon fibre angle could be measured at 71.91 and 83.40% of distance measurement points across all quantitatively analysed sample sections, for inserting and approaching fibres, respectively. Exclusions were made due to cortical bone loss, haphazard degenerated fibres or poor fibre definition, preventing accurate or reliable assessment. ICCs were 0.91 (0.87–0.93) for all angle measurements made, 0.82 (0.75–0.87) for intersecting fibres and 0.80 (0.74–0.84) for approaching fibres, and, for separate distance measurement points, ranged from 0.86 (0.78–0.91) at the 40% point to 0.94 (0.90–0.96) at the 20% point.

The overall mean angle of inserting fibres was 30.05 ± 0.72^o^. Averaged across all enthesis distance measurement points (Table 2), there were no significant differences between individual fingers, or fingers of combined sexes, although a significant difference of 4.55^o^ (*p* < 0.05) was present between sexes. Inserting fibre angles were similar across all distance measurement points, ranging from 27.69 ± 1.51^o^ (80% point) to 33.05 ± 1.47^o^ (40% point) for overall data (all measurements reported in Table S-2), with no significant difference existing between distance points within finger or sex groupings. The angle of approaching fibres averaged 15.20^o^ overall, and all measurements (reported in Table S-3) were more acute than their inserting fibre counterpart, describing a widening of angle as fibres came to insert. This angle change exhibited significant variability along the enthesis length. The greatest angle change was present at the 20% enthesis distance point (21.05 ± 1.47^o^) significantly wider than the angle change at the 50, 60 and 80% points by 8.41^o^ (*p* < 0.001), 9.21^o^ (*p* < 0.001) and 7.34^o^ (*p* < 0.01), respectively, for overall data (Fig. 5e).

**Table 2:** Angle (^o^) of Inserting Fibres Averaged Across All Distance Measurement Points

Finger	Male	Female	***All***
**Index**	26.77 (1.85)	33.96 (1.85)	30.37 (1.30)
**Middle**	27.98 (2.03)	31.37 (1.85)	29.62 (1.35)
**Ring**	28.42 (1.85)	31.51 (1.85)	29.96 (1.30)
**Little**	28.07 (1.85)	32.44 (2.03)	30.26 (1.35)
***All***	27.78 (1.01)	32.33 (1.01)	30.05 (0.72)

Mean (± standard error)

## Discussion

FDP avulsion injury incurs considerable functional morbidity and outcomes after surgical repair are often poor [1, 8–10]. The enthesis at the tendon-bone interface is naturally designed with adaptations for optimum function and damage prevention, not satisfactorily restored through current surgical methods. This study has examined key structural features of the native enthesis as a guide to developing a novel tissue engineered replacement and to potentially enhance current surgical techniques. Results revealed a consistent trapezoidal insertion shape, with significant size differences primarily influenced by smaller little finger dimensions, and a fibrocartilaginous enthesis with uniform inserting tendon fibre angle but regional variation in fibrocartilage content and change in tendon fibre angle. The implications of these findings on biomechanics, ITE design and surgical repair are considered within their macroscopic and microscopic context.

Macroscopically, the consistency of the trapezoidal insertion suggests biomechanical advantages to the shape. The FDP insertion is at risk of avulsion due to a powerful muscle belly combined with narrow attachments to effect strong but precise movement. The distal FDP tendon has a flattened oval shape in cross section, but flaring out to a trapezoidal insertion fills the wide base and narrowing proximal shaft on the DP flexor surface, maximising tendon-bone contact surface area to spread stress force whilst retaining function-specific positioning. Repetition of this insertion shape over all fingers, as well as acting as a further avulsion risk reducing mechanism by distributing muscle force over multiple tendon attachments [13], implies that the shape also favourably balances increased surface area with minimal areas of stress concentration. Recreation of the trapezoidal interface shape should therefore be a key aim for ITE design and surgical repairs.

A strong repair of a trapezoidal insertion may be achieved by 3 point fixation: 2 at the insertion base and 1 at the apex. Such an arrangement for an FDP repair can be fashioned with 2 micro bone anchors at the insertion base corners and a pull-out suture at the apex, favoured by certain authors as a strong and reliable repair [2, 36]. Although some surgeons may consider that this technique increases technical complexity and potential for complications, it optimally reconstructs an important morphological and biomechanical feature, as well as providing an ultimate tensile strength similar to the native insertion [36]. For tissue engineers, the challenge is to construct and maintain the trapezoidal interface, between engineered tendon and bone components which may be of either fixed or variable form during in vitro culture. Fixed form scaffolds (e.g. bone ceramics) require precise fabrication to achieve the specific interface area, whilst scaffolds varying in shape during culture (e.g. contracting hydrogels) may need morphological manipulation. Formation and culture using either scaffold type demands detailed design specifications, for example for 3-D printed molds, culture well inserts or bioreactors, based on the morphometric footprint data in Table 1.

Table 1 serves as a size guide for constructing the trapezoidal FDP insertion, for a single average size or multiple sizes based on a particular sex and finger. Surgically this information may be especially valuable in chronic, neglected avulsions or complex revisions demanding a tendon graft and no DP footprint haematoma is visible. The significant size variability for height and base width dimensions indicates that a universal size approach may not be appropriate surgically or for translatable ITE designs. Equally, 8 different sizes differing by fractions of millimetres is not practical or resourceful. When considering meaningful size differences, the major trends in significantly different data may provide the best approach. With all but 1 significant size differences between fingers involving the little finger (surface area, height, base width), the little finger alone might be categorized as one size level lower. Due to smaller female dimensions compared to males [surface area, height (significantly), base width] it may also be appropriate to group the lower level male size (little finger) with the higher level female size (index, middle, ring finger) as their means are also similar. The lower level female size (little finger) then stands as a separate category. Averaging data in Table 1 within these categories to the nearest millimetre establishes a 3-level size guide (Table 3) that may optimally balance variability with practicality, based on pragmatic whole millimetre designs.

**Table 3:** 3-Level Size Guide Dimensions (mm) for Trapezoidal FDP Insertion

Size	Height	Base Width	Apex Width
**Large**	6	9	2
**Medium**	5	8	2
**Small**	4	7	2

*Large* male index, middle, ring finger. *Medium* male little finger; female index, middle, ring finger. *Small* female little finger

Microscopically, the fibrocartilaginous nature of the FDP enthesis was confirmed. This classification was expected due to the FDP insertion position, near the DIPJ, since attachment to bony epiphyses and apophyses is characteristic of fibrocartilaginous entheses [13, 19, 37]. Tendon fibres of fibrocartilaginous entheses are classically described as crossing the tidemark at approximate right angles [11, 37, 38], whereas fibres in areas lacking fibrocartilage attach to bone at acute angles [37]. The finding that inserting FDP fibres, measured intersecting the tidemark, averaged 30.05 ± 0.72^o^ aligns with the observation that, although classified as fibrocartilaginous overall, substantial areas of the enthesis lacked distinguishable fibrocartilage and were fibrous.

Fibrocartilage appeared to predominate in the proximal enthesis region, agreeing with previous histological reports of fibrocartilage concentrated in the tendon portion nearest the joint it crosses [37, 39]. Furthermore, the tendon fibre angle change was significantly widest at the most proximal enthesis distance point measured (20% length), implying that this proximal region is of particular biomechanical importance. UF presence mitigates against the shear force produced by these proximal fibres undergoing a wider angle change before insertion by promoting gradual fibre bending [11, 18, 26–28]. UF in particular also protects against compression forces [12, 18, 40], demonstrating likely compression of the more proximal (deeper) FDP fibres by more distal (superficial) fibres during DIPJ movement. CF relates to the degree of tendon loading on the bone [18, 26, 27], suggesting greater force transmission through these proximal/deeper tendon fibres, which may also explain the frequent finding of a convex bony profile at the enthesis, with peak elevation inclined more proximally, deformed by the more proximal fibres. Investigation of whether chronic degeneration or acute avulsion begins more or less frequently in the proximal region would divulge the level of protection afforded by the fibrocartilage in this vulnerable area.

The foremost implications on FDP ITE design and surgical avulsion repair from the microscopic findings are establishing a fibrocartilaginous interface with an overall approximate insertion angle of 30^o^. Since the fibrocartilaginous tissue zones are not regenerated in surgical repair [20–23] this confirms the importance of ITE research at the FDP insertion, both for in vivo repair augmentation and in vitro models. Promising in vivo studies promoting fibrocartilage formation and mechanical properties at repair sites, for example with cellular therapy or biochemical modulation, remain primarily in the animal model stage [41, 42] whilst in vitro models are not morphologically specific to a particular enthesis. Before employing strategies to establish the fibrocartilage transition, throughout the entire interface or regionally, enthesis specificity can be enhanced by recreating the native tendon fibre insertion angle, and consequently the local biomechanics. For an FDP ITE model, this may be encouraged by a 30^o^ angle between tendon and bone components, incorporated into the design of molds, culture well inserts or bioreactors in conjunction with the trapezoidal interface.

Surgically, the angle of FDP fixation onto the DP varies with repair technique. Considering the most common techniques, in the standard pull-out button repair, sutures holding the tendon are drawn through an oblique anterograde DP drill hole for tying over the nailplate, whereas bone anchors holding the tendon are typically placed retrograde into the DP at 45^o^. Both aim to achieve secure tendon-bone contact, however it is unknown whether different insertion angles impact upon the direction of inserting tendon fibres once the healing attachment has matured and collagen fibres have realigned. From an anatomical standpoint, encouraging a 30^o^ (anterograde) insertion of tendon fibres is optimal, most relevant to consider for drill angle when creating passage through the DP for the pull-out button repair sutures, as long as nailplate exit distal to the germinal matrix and lunula is maintained. Selection of bone anchor angle is primarily related to pull-out strength and avoidance of cortical penetration, however, although retrograde placement is classically viewed as the most biomechanically favourable [43], anterograde angles have also been shown to give the same or greater load to failure [44, 45], possibly reflecting the more native insertion angle.

The limitations of this study are primarily related to the sample and measurement methodologies. Results aimed to provide population data descriptions, but were taken from a local Scottish sample with an age range (65–95) most likely older than the average age of patients with FDP avulsion. Later age is associated with histopathological enthesis changes such as microtears and microdamage [46], however the tendon fibre angle measurement methodology mitigated against this by analysing multiple enthesis regions and excluding degenerated areas. The shape and size of the FDP enthesis is unlikely to change over time in healthy individuals, with results translatable to younger populations, but size variation may exist due to local genetics or other variables unknown to this sample such as height, body mass index or cumulative manual activity level. Sample size was determined through similar and improved numbers from other cadaveric FDP insertion studies [29, 47], and although many significant differences were found across sex and finger groups in the limited sample, data interpretation has deliberately focused on the larger differences or recurring trends.

The measurement methodologies used were subjective, but were based on published techniques [25, 29, 35] and ICCs for all but 1 data set showed ‘excellent’ or ‘good’ reliability [48]. Apex width of the FDP footprint was the least reliable measurement, suggesting subjective rounding to the apex of the trapezoidal shape where determination of a horizontal measurement was difficult. Apex width measurements were small (range 1.33–2.11 mm) and reliability of internal angle trapezoid measurements was excellent (ICC 0.99), implying that inter-observer variability was unlikely to impact meaningfully on trapezoid dimensions. However it is acknowledged that morphometrics, including tendon fibre angle, were linear measurements describing imperfectly straight lines, but were applied to extract useful, relatable data. Measurements were also 2-D representations of 3-D structures. Although the FDP footprint has a relatively flat profile for analysis, histological analysis was only undertaken in mid-sagittal section. Results from para-sagittal planes may have varied, however mid-sagittal was expected to be the optimal analysis plane since the central enthesis region contains the most organised collagen fibres [49] and most complex arrangement of fibrocartilaginous layers [50].

## Conclusion

In summary, this study his examined the native macroscopic and microscopic anatomy of the FDP enthesis, to gain greater morphological and biomechanical understanding of an important and commonly injured tendon-bone interface that may benefit from enhanced or novel treatments. The findings are distilled as potential recommendations to hand surgeons and guides to interfacial tissue engineers for recreating the native insertion, and highlight region specific anatomical knowledge as the key to establishing translatable ITE models. These investigations may be applied to entheses in any body region, similarly providing the foundation to develop superior therapeutic options for a wide range of debilitating musculoskeletal injuries.

## Supplementary Information

**Additional file 1 Fig. S1** FDP Footprint Image Analysis. **a)** Lower half of stained DP with unstained FDP footprint. *Dotted line* shows mapped FDP footprint perimeter, *full line* shows consequent bounding box, processed to create the binary mask footprint image within its bounding box [**(b)** and **(c)**]. Footprint surface area is quantified from the area inside the footprint perimeter. **b)** General footprint measurements. Base width (*BW*) is the widest measurement (i.e. width of bounding box), and apex width (*AW*) is the highest point at which the sloping sides turn horizontally towards the midline. Height is calculated as the mean of height at mid-width of the bounding box (*H*_*1*_) and maximum height (*H*_*2*_, i.e. height of bounding box). **c)** 4 internal angles (apex left, *AL*; apex right, *AR*; base left, *BL*; base right, *BR*) are calculated as a mean of 2 trapezoids (subscripts *1* and *2)*. The base of the trapezoids are defined by the perpendicular at the highest point of left or right bounding box intersection (subscript *1*, *dotted line*) or at the lowest point of the footprint (subscript *2*, *broken line*). Apices are positioned the same for both trapezoids. **Table S1.** Internal Angles (^o^) of FDP Footprint Trapezoid. Results are presented as Mean (± standard error). **Table S2.** Angle (^o^) of Inserting Fibres at Enthesis Distance Measurement Points. **Table S3.** Angle (^o^) of Approaching Fibres at Enthesis Distance Measurement Points. Results are presented as Mean (± standard error).

## Data Availability

The datasets used and/or analysed during the current study are available from the corresponding author on reasonable request.

## Abbreviations

FDP: Flexor digitorum profundus
DP: Distal phalanx
