# Supplementary material for: Anatomy and histomorphology of the flexor digitorum profundus enthesis: functional implications for tissue engineering and surgery
Source: BMC Musculoskelet Disord. 2021 Dec 10;22:1032. doi: 10.1186/s12891-021-04922-1 (PMC8665545; doi:10.1186/s12891-021-04922-1)
Supplement: Supplementary file 1 — Additional file 1 Fig. S1 FDP Footprint Image Analysis. a) Lower half of stained DP with unstained FDP footprint. Dotted line shows mapped FDP footprint perimeter, full line shows consequent bounding box, processed to create the binary mask footprint image within its bounding box [(b) and (c)]. Footprint surface area is quantified from the area inside the footprint perimeter. b) General footprint measurements. Base width (BW) is the widest measurement (i.e. width of bounding box), and apex width (AW) is the highest point at which the sloping sides turn horizontally towards the midline. Height is calculated as the mean of height at mid-width of the bounding box (H1) and maximum height (H2, i.e. height of bounding box). c) 4 internal angles (apex left, AL; apex right, AR; base left, BL; base right, BR) are calculated as a mean of 2 trapezoids (subscripts 1 and 2). The base of the trapezoids are defined by the perpendicular at the highest point of left or right bounding box intersection (subscript 1, dotted line) or at the lowest point of the footprint (subscript 2, broken line). Apices are positioned the same for both trapezoids. Table S1. Internal Angles (o) of FDP Footprint Trapezoid. Results are presented as Mean (± standard error). Table S2. Angle (o) of Inserting Fibres at Enthesis Distance Measurement Points. Table S3. Angle (o) of Approaching Fibres at Enthesis Distance Measurement Points. Results are presented as Mean (± standard error). [file 12891_2021_4922_MOESM1_ESM.docx]

**Supplementary information**

**Figure S1**


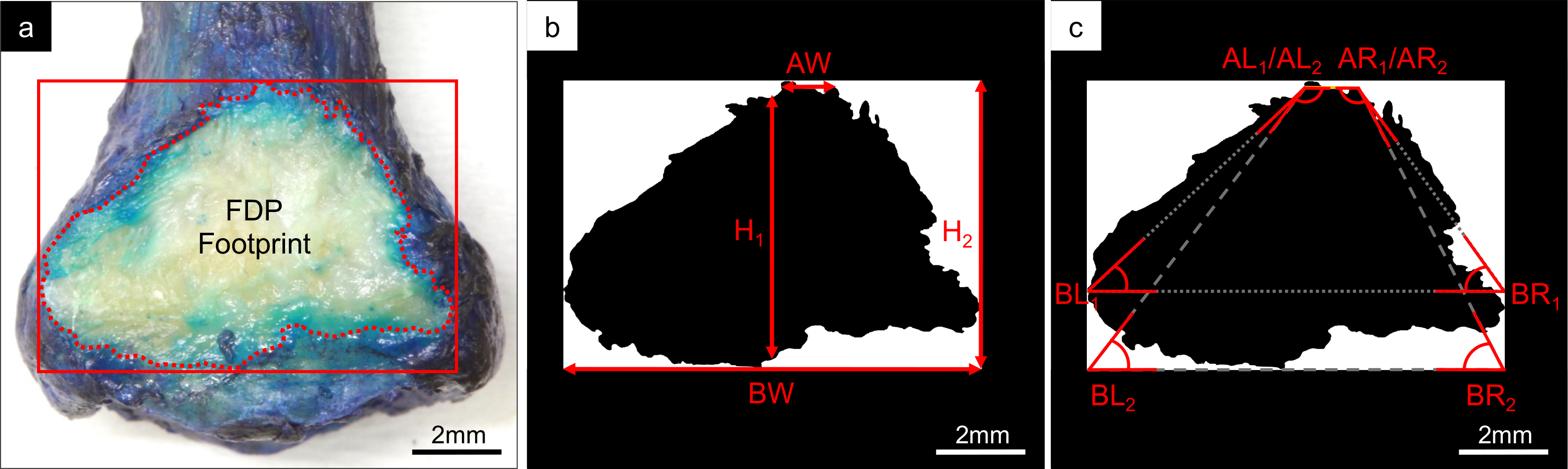


**Figure S1. FDP Footprint Image Analysis**

**a)** Lower half of stained DP with unstained FDP footprint. *Dotted line* shows mapped FDP footprint perimeter, *full line* shows consequent bounding box, processed to create the binary mask footprint image within its bounding box [**(b)** and **(c)**]. Footprint surface area is quantified from the area inside the footprint perimeter. **b)** General footprint measurements. Base width (*BW*) is the widest measurement (i.e. width of bounding box), and apex width (*AW*) is the highest point at which the sloping sides turn horizontally towards the midline. Height is calculated as the mean of height at mid-width of the bounding box (*H_1_*) and maximum height (*H_2_*, i.e. height of bounding box). **c)** 4 internal angles (apex left, *AL*; apex right, *AR*; base left, *BL*; base right, *BR*) are calculated as a mean of 2 trapezoids (subscripts *1* and *2)*. The base of the trapezoids are defined by the perpendicular at the highest point of left or right bounding box intersection (subscript *1*, *dotted line*) or at the lowest point of the footprint (subscript *2*, *broken line*). Apices are positioned the same for both trapezoids.

**Table S-1.** Internal Angles (^o^) of FDP Footprint Trapezoid

| **Finger** | **Base** | | **Apex** | |
| --- | --- | --- | --- | --- |
|  | **Left** | **Right** | **Left** | **Right** |
| **Male** |  |  |  |  |
| **Index** | 57.88 (3.02) | 60.02 (2.09) | 122.14 (3.02) | 119.91 (2.09) |
| **Middle** | 56.50 (3.02) | 53.86 (2.09) | 123.49 (3.02) | 126.14 (2.09) |
| **Ring** | 54.50 (3.22) | 55.02 (2.28) | 125.51 (3.23) | 124.96 (2.28) |
| **Little** | 52.48 (3.02) | 52.65 (2.09) | 127.55 (3.02) | 127.50 (2.09) |
| ***All*** | 55.36 (2.17) | 55.43 (1.22) | 124.65 (2.17) | 124.58 (1.22) |
| **Female** |  |  |  |  |
| **Index** | 54.60 (3.02) | 55.32 (2.09) | 125.38 (3.02) | 124.70 (2.09) |
| **Middle** | 50.18 (3.02) | 52.95 (2.09) | 129.83 (3.02) | 127.08 (2.09) |
| **Ring** | 51.69 (3.02) | 55.79 (2.09) | 128.29 (3.02) | 124.17 (2.09) |
| **Little** | 50.41 (3.02) | 53.56 (2.09) | 129.62 (3.02) | 126.53 (2.09) |
| ***All*** | 51.72 (2.15) | 54.41 (1.20) | 128.28 (2.15) | 125.62 (1.21) |
| **Collective** |  |  |  |  |
| ***All*** | 53.54 (1.53) | 54.92 (0.85) | 126.47 (1.53) | 125.10 (0.86) |

Mean (± standard error).

**Table S-2.** Angle (^o^) of Inserting Fibres at Enthesis Distance Measurement Points

| **Finger** | **20%** | | | **40%** | | | **50%** | | |
| --- | --- | --- | --- | --- | --- | --- | --- | --- | --- |
|  | **Male** | **Female** | ***All*** | **Male** | **Female** | ***All*** | **Male** | **Female** | ***All*** |
| **Index** | 28.08  (5.15) | 43.60  (5.95) | 34.53  (3.96) | 26.97  (3.20) | 36.14  (3.20) | 31.56  (2.17) | 28.70  (3.85) | 29.27  (4.30) | 29.08  (2.74) |
| **Middle** | 29.08  (5.42) | 36.67  (4.95) | 32.96  (3.72) | 34.56  (4.53) | 39.43  (5.55) | 37.16  (3.37) | 26.44  (4.97) | 29.55  (4.30) | 28.05  (3.11) |
| **Ring** | 27.61  (5.79) | 31.65  (4.95) | 29.23  (3.82) | 30.99  (3.92) | 36.83  (4.53) | 33.96  (2.85) | 30.07  (3.85) | 31.18  (3.85) | 30.63  (2.59) |
| **Little** | 30.32  (4.95) | 26.80  (6.63) | 31.16  (3.97) | 27.01  (3.20) | 28.20  (7.84) | 29.52  (3.02) | 28.75  (3.85) | 34.66  (6.09) | 30.93  (3.16) |
| ***All*** | 28.77  (4.36) | 35.18  (4.42) | 31.97  (3.09) | 29.77  (1.77) | 36.33  (2.31) | 33.05  (1.47) | 28.54  (1.96) | 30.80  (2.16) | 29.67  (1.45) |
| **Finger** | **60%** | | | **80%** | | |  |  |  |
|  | **Male** | **Female** | ***All*** | **Male** | **Female** | ***All*** |  |  |  |
| **Index** | 25.25  (3.12) | 28.49  (3.12) | 26.87  (2.27) | 22.42  (4.02) | 32.95  (3.60) | 28.04  (2.65) |  |  |  |
| **Middle** | 25.58  (2.79) | 25.45  (2.79) | 25.51  (2.03) | 30.39  (4.02) | 30.38  (3.29) | 29.97  (2.52) |  |  |  |
| **Ring** | 25.10  (2.79) | 31.61  (2.79) | 28.35  (2.03) | 26.14  (3.60) | 28.56  (3.29) | 27.27  (2.39) |  |  |  |
| **Little** | 29.34  (2.79) | 42.17  (3.60) | 34.81  (2.29) | 22.95  (8.05) | 27.67  (4.65) | 25.46  (4.03) |  |  |  |
| ***All*** | 26.27  (1.48) | 31.50  (1.57) | 28.89  (1.08) | 25.62  (2.26) | 29.75  (1.83) | 27.69  (1.51) |  |  |  |

Mean (± standard error).

**Table S-3.** Angle (^o^) of Approaching Fibres at Enthesis Distance Measurement Points

| **Finger** | **20%** | | | **40%** | | | **50%** | | |
| --- | --- | --- | --- | --- | --- | --- | --- | --- | --- |
|  | **Male** | **Female** | ***All*** | **Male** | **Female** | ***All*** | **Male** | **Female** | ***All*** |
| **Index** | 14.21  (3.34) | 13.85  (4.01) | 14.52  (2.60) | 16.56  (2.63) | 18.35  (2.63) | 17.48  (1.79) | 15.52  (2.06) | 19.60  (2.22) | 17.56  (1.44) |
| **Middle** | 9.86  (3.66) | 10.65  (3.34) | 10.13  (2.53) | 14.85  (2.97) | 17.57  (2.78) | 16.08  (1.96) | 11.89  (2.80) | 18.79  (2.22) | 15.82  (1.67) |
| **Ring** | 4.87  (4.48) | 11.06  (3.34) | 9.03  (2.67) | 14.31  (2.97) | 21.52  (3.04) | 17.86  (2.06) | 15.68  (2.06) | 17.59  (2.22) | 16.73  (1.44) |
| **Little** | 10.02  (3.34) | 12.90  (3.66) | 11.41  (2.53) | 13.61  (2.63) | 19.63  (3.23) | 16.29  (1.96) | 18.06  (2.22) | 21.05  (2.48) | 19.56  (1.58) |
| ***All*** | 10.21  (3.07) | 12.34  (3.06) | 11.27  (2.16) | 14.90  (2.01) | 18.95  (2.05) | 16.92  (1.43) | 15.55  (1.30) | 19.28  (1.30) | 17.42  (0.92) |
| **Finger** | **60%** | | | **80%** | | | **Average** | | |
|  | **Male** | **Female** | ***All*** | **Male** | **Female** | ***All*** | **Male** | **Female** | ***All*** |
| **Index** | 14.48  (1.85) | 16.96  (1.85) | 15.71  (1.33) | 12.45  (2.44) | 13.25  (2.24) | 12.85  (1.63) | 14.93  (1.73) | 16.86  (1.73) | 15.89  (1.21) |
| **Middle** | 11.06  (1.85) | 16.84  (1.85) | 13.92  (1.33) | 15.45  (2.44) | 13.61  (2.10) | 14.30  (1.57) | 12.08  (1.82) | 15.10  (1.73) | 13.64  (1.24) |
| **Ring** | 16.22  (1.85) | 14.92  (1.85) | 15.57  (1.33) | 13.86  (2.24) | 16.27  (2.10) | 15.18  (1.52) | 14.65  (1.73) | 15.66  (1.73) | 15.16  (1.21) |
| **Little** | 20.30  (1.74) | 20.01  (1.85) | 20.25  (1.38) | 13.92  (2.24) | 13.41  (2.10) | 13.62  (1.52) | 14.88  (1.73) | 17.31  (1.73) | 16.10  (1.21) |
| ***All*** | 15.56  (1.31) | 17.17  (1.32) | 16.36  (0.93) | 13.86  (1.57) | 14.12  (1.51) | 13.99  (1.09) | 14.16  (1.38) | 16.23  (1.37) | 15.20  (0.97) |

Mean (± standard error).
